# Childhood obesity management shifting from health care system to school system: intervention study of school-based weight management programme

**DOI:** 10.1186/1471-2458-14-1128

**Published:** 2014-11-03

**Authors:** Albert Lee, Mandy Ho, Vera MW Keung, Amy CM Kwong

**Affiliations:** Centre for Health Education and Health Promotion, JC School of Public Health and Primary Care, The Chinese University of Hong Kong, 4th Floor, Lek Yuen Health Centre, Shatin, New Territory Hong Kong; The Children Hospital at Westmead Clinical School, University of Sydney, Sydney, Australia

**Keywords:** Childhood obesity, School based programme, Intervention

## Abstract

**Background:**

Home and school environments conducive for unhealthy eating and physical inactivity are precursors of obesity. The aim of this study is evaluation of the effectiveness of a multi-component school-based weight management programme for overweight and obese primary school children via a home-school joint venture.

**Methods:**

This study made use of variety of behavioural modification strategies integrating into the Health Promoting School approach to promote healthy lifestyles. The participants were overweight and obese students aged between 8 and 12 from six participating schools. The interventions involved students attending ten 75 minutes after-school sessions and one 3-hour week-end session of practical interactive and fun activities on healthy eating and exercise, and meal plan together with parents and printed tailor-made management advices. Parents received an introductory seminar with 2 sets of specially designed exercise for their overweight children. The tools to measure bodyweight and fat percentage and standing height were bio-impedance body fat scale and a portable stadiometer. Self-administered questionnaire was used to measure knowledge, attitudes and behaviours. McNemar test was utilized to compare the proportions of behaviour changes within the same group to assess for the trends of changes. BMI z-score and body fat percentage of intervention participants at baseline, 4 month and 8 month were compared pair-wisely using tests of within subject contrasts in repeated measures ANOVA to assess for programme sustainability.

**Results:**

Those students in the intervention group reduced their BMI z-score (-0.21, 95% CI -0.34 to -0.07, P = 0.003) and body fat (-2.67%, 95% CI -5.12 to -0.22, P = 0.033) compared to wait list control group with statistical significant, and the intervention group also had a significant reduction in BMI z-score (-0.06, 95% CI -0.11, -0.007, P = 0.028) and body fat (-1.71%, 95% CI, -3.44 to 0.02, P = 0.052) after a 4 month maintenance period. Improvement of dietary habits and positive attitudes towards exercise were observed among the intervention group.

**Conclusion:**

School based weight management programme integrated into a Health Promoting School approach with improved school policies and environment in supporting individual skills of obese students and their parents appears to be a promising practice for sustaining weight control.

**Trial registration:**

ISRCTN58795797.

**Electronic supplementary material:**

The online version of this article (doi:10.1186/1471-2458-14-1128) contains supplementary material, which is available to authorized users.

## Background

Prevalence of childhood obesity is rising globally and accelerates at faster rate among countries undergoing rapid economic development [1, 2]. The prevalence of overweight and obesity among Chinese population especially mainland China, is fast catching up with the West [3]. There are already signs of epidemic of childhood obesity in Hong Kong, Taiwan and Macao [4, 5]. Another emerging country, Brazil also shows similar pattern with increase from 4% to 14% between 1974 and 1997 [6] with a shift towards the poor [7]. Childhood obesity poses immediate health concerns and long-term health risks [8], and social, emotional and psychological difficulties [9]. Obesity is now a significant risk factor accounting for the global burden of diseases [10]. It also poses problems in learning as shown by Iceland study that body mass index, diet and physical activity would explain up to 24% of the variance in academic performance after controlling major confounders [11].

A recent systematic review has reported that lifestyle interventions incorporating dietary and exercise components with or without behaviour therapy would lead to improvement of weight and cardio-metabolic outcomes among children [12]. Only two studies on Chinese population were included. One study was conducted in one middle school in Beijing China with 33 students receiving family based behavioural treatment and 35 students in control group [13], and the other study in Taiwan with 12 weeks heart health education and physical activity program [14].

Although general practitioners (GPs) as the first point of patient contact should be able to tackle the issue of childhood obesity, lack of experience, time and resources as well as guidelines on practical approach become the barriers [15]. Individualized interventions by GPs might not be intensive enough to bring about weight reduction for substantial behavioural modification [16, 17]. Meta-analytic review of 64 obesity prevention programmes (46 trials) for children and adolescents did not reveal significant effects to prevent weight-gain [18]. Community based intervention with greater emphasis on environmental changes (both physical and social) has shown to be more effective as children’s behaviours are much more environmental dependent [19, 20]. Parental or family involvement in interventions would have impact on nutrition and physical activities of the children [21, 22] leading to weight reduction [23].

School is another setting in which the children spend a substantial amount of time. Policies targeting the school environment can be considered as key strategy to address childhood obesity as school resources and practices would have impact on the availability of specific food and beverages [24]. Evidence has shown that home and school environments promoting unhealthy eating habit and physical inactivity are precursors of obesity [25]. Systematic reviews on school based obesity prevention programmes did not provide consistent evidence of the efficacy of school-based programme [18, 23, 26, 27]. Most of the interventions focused on short-term changes right after the intervention [27–29], or process outcomes with no significant changes of behavioural outcomes [27, 30, 31]. Despite major reduction in consumption of high calorie beverages and snacks among intervention group in a randomised school-based intervention involving families and teachers to prevent excessive weight gain among adolescents in Brazil, no significant change of BMI was observed [32].

‘Healthy Setting’ approach such as Health Promoting School (HPS) delivers health promotion activities in the context of daily life and provides the ‘social structures’ to reach the defined population i.e., the students [33–36]. Schools adopting the HPS approach would be more willing to implement more intensive health promoting activities for the overweight and obese students so it is a potential avenue for more rigorous multi-component programme. This pilot study employed measures to evaluate the efficacy and sustainability of a 4 month school-based lifestyle intervention integrating into a boarder HPS approach on weight loss and behavioural changes in overweight and obese students by fostering individual focused weight management plan. This study also evaluated the impact of the interventions on their parents. To our knowledge, this is one of the very few studies delivered in schools which implemented a broader HPS initiative.

## Methods

### Ethics statement

The study has been registered with International Standard Randomised Controlled Trial Number Register (ISRCTN 58795797) and the study was vetted and approved by Sub-committee of Health Care Promotion Fund of Hong Kong SAR Government and recommended to Health Care Promotion Fund. The project also met the safety and ethical requirement of University. Inform parental consent was obtained and investigations were conducted according to the principles expressed in the declaration of Helsinki. The study was endorsed by the Chinese University of Hong Kong Faculty of Medicine Survey and Behavioural Research Ethics (SBER) Sub-committee, and approval was granted by University SBER to conduct survey on observation of human behaviours.

### Study design and setting

The study was designed as a school-based intervention study adopting wait-list control approach over one academic year from August 2007 to July 2008. Invitation letters were sent to 65 primary schools participating in HPS project. Sixteen schools replied and based on the prevalence of obesity children in the school, school district, readiness of the school, and also similarity in socio-demographic background among participating schools, 6 schools were selected to participate. The schools were responsible to recruit eligible students for the program. The inclusion criteria were age 8 to 12 years, overweight or obese according to International Task Force definitions [37] with parents’ commitment to participate in the home-school joint venture. Informed written consent was obtained from parents prior to joining the project.

In Hong Kong, all students undergo anthropometric measurements annually with results recorded so teachers would identify overweight subjects. Teachers in Hong Kong are quite well equipped with skills in discussing sensitive issues such as overweight. They are quite comfortable to discuss the issues with students and parents if there is structured educational programme to help them emphasising positive living rather than negative images.

**Figure 1:**
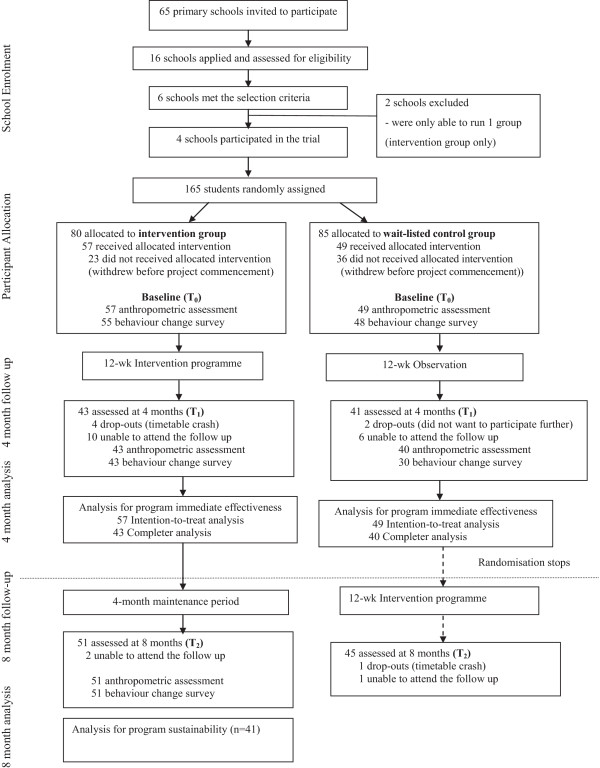
**Study design and participant flow through the study.**

Within the school, those eligible subjects consented to participate were randomly assigned (random numbers) to start the program immediately (intervention group) or received the intervention 4 months later (wait-list control group). Due to difficulties in school administrative arrangement, only 4 primary schools were able to undertake both intervention and control groups while the other 2 schools only had students in the intervention group. The findings reported in this paper were based on data from the 4 schools with both intervention and control groups. Figure 1 illustrates the study design and participant’s flow.

### Interventions

This study piloted a tailor-made weight management plan for those overweight and obese children using combination of behavioural, dietary and physical activity interventions in school setting as well as home setting. The project team guided the participating schools to implement series of school-based activities promoting healthy eating and active lifestyles throughout the project period under the broad HPS approach enhancing the awareness of health lifestyle for all students (Additional file 1). This approach would reduce the contamination effect for the intervention group of students as they received the tailor-made weight management programme as well as whole school approach health promotion activities. It also reduced the co-interventions of students in wait-list control group as all students were involved in the general school based health promotion activities and they enrolled during second phase. This study evaluated the effectiveness of this multi-component weight management programme.

#### Intervention program for overweight and obese students

Intervention group received 4 months intensive intervention conducted by the dietician, nutritionist and physiotherapist of the project team as well as teachers of the participating schools followed by 4 months maintenance phase. The program consisted of ten 75-minutes after-school sessions and 3 hours weekend session of practical, interactive and fun activities covering the topics on healthy eating, exercise and positive self-image. The sessions included 5 nutrition education sessions, 1 session on body image and self-esteem, 1 session on sport safety and 4 sessions of supervised physical activity (Table 1). Strategies such as assessing readiness for change, goal-setting, self-monitoring, problem-solving, role-playing, motivational reinforcement and awards were adopted to enhance behavioural modification. Teaching strategies included interactive games, practical workshops, problem-solving activities, fun-based physical activity. A meal plan (1500 to 1800 kcal/day according to their age and sex, 50 to 55% total energy from carbohydrate, 15 to 20% from protein and 30% from fat) was provided for all participants in the presence of their parents at the start. Students also received printed tailor-made weight management advices. The dietetic advice included suggestions on portion of core food groups and snacks; food selection and healthy eating strategies. Exercise plans consisted of prescription on aerobic, stretching and strengthening exercise as well as suggestions on strategies to be more physical active.

**Table 1:** **Weight management intervention session topics**

Session	Topics	Session conducted by
1	Goal setting and introduction of serve sizes	Nutritionist/dietician
2	Exercise and energy expenditure, sport safety	Physiotherapy
3	Supervised physical activity session I*	School PE teachers
4	Body image and self esteem	School teacher
5	Energy balance and smart tips for weight control	Nutritionist/dietician
6	Supervised physical activitysession II*	School PE teachers
7	Healthy snack	Nutritionist/dietician
8	Label reading	Nutritionist/dietician
9	Supervised physical activity session III*	School PE teachers
10	Smart dinning out	Nutritionist/dietician
11	Supervised physical activity session IV*	School PE teachers

*Fun-based physical activity session, lesson plans were designed by school physical education (PE) teachers with guidance from the project physiotherapist.

#### Involvement of parents

Parents of intervention group received an introductory seminar on the basic principles, skills and knowledge on weight management at the beginning of the programme. Two sets of exercise specially designed for overweight children were introduced to parents together with their children so they would practise together at home. Parents received two sessions of 1-hour follow-up on further skills about healthy eating and exercise strategies to assist weight control in children. During the follow-up sessions, the dietician and physiotherapist discussed with parents in group to monitor students’ progress at home and provided solutions for problems encountered. Parents were also invited to join the food label education training together with their children. Results of anthropometric measurement together with dietary and excise advice were explained to parents together with their children immediately after each assessment to enhance their awareness. Parents were encouraged to convert their enquiries to the project team via school teachers if they could not attend the planned workshop or face-to-face consultation. Student handbook contained a designated section that required parents to give weekly comments on their children’s progress.

### Outcome measures

Outcome measures were assessed at baseline (T_0_) before the intervention, and completion of 4 months intervention (T_1_) to evaluate the effectiveness of program and 8 months from baseline, T_2_ (after the 4-months maintenance period) to evaluate the sustainability of the program. The primary study outcomes were age- and sex-adjusted Body Mass Index z-score (BMI z-score) and body fat percentage. Secondary outcomes included self-reported attitude and behavioural variables.

#### Anthropometry

Body weight (to nearest 0.1 kg) and body fat percentage (to nearest 0.5%) were measured by bio-impedance body fat scale (Model: TBF-521, Tanita, Japan) with participants lightly clothed without shoes. Standing height (to the nearest cm) was measured with a portable stadiometer (Model: 214, SECA). Data collection was performed by trained personnel who were blinded to group allocation and were not involved in conducting sessions for the students. BMI z-score was calculated based on age- and sex-specific reference value [38].

#### Attitude and behavioural changes

Students completed a set of questionnaire adopted from other studies designed for school children with a total of 20 items to investigate the attitude towards healthy eating and exercise, dietary behaviour, participation in physical activity, self-control, self-perception of weight and weight management practice [5, 21, 22]. Data were collected by trained staff reading out the questions and explaining some difficult concepts such as portion sizes of food and exercise intensity. They were also blinded to participant group allocation.

Parents completed a self-administered questionnaire on the general health condition, eating and exercise habit of their children, cooking methods used at home, frequencies of engaging in different physical activities (such as housework, outings and sports activities) with their children. The questionnaire had undergone face and content validation by pilot testing on group of parents and consultation with experts. Additional file 2 lists the contents of the questionnaires.

#### Program evaluation

Programme evaluation was conducted by survey completed by students, parents and teachers at the end of the project to collect their feedbacks.

### Sample size considerations

Taken reference from previous study [22] and the assumption that 15% of students who had healthy eating habits (π_0_ = null hypothesis proportion), would increase to 30% after intervention (π = proportion of interest), the sample size for each group should be 52 (N = 104) giving a power of 80% (u = 0.84) and level of significance at 5% (v = 1.96).


### Statistical analysis

All data were tabulated and analysed by the SPSS package version 11.5 (SPSS, Inc) and the level of significance was set at 0.05. Chi-square test and independent *t*-test were used to assess for between-group differences in intervention and control groups during baseline. As recommended by the CONSORT guidelines for randomized trials [39], the primary analysis was performed on an intention-to-treat (ITT) basis, with all participants included in the analysis according to original group allocation using linear mixed models to evaluate group differences in BMI z-score and body fat percentage at baseline and 4 months. Completer analysis was performed by the repeated measures analysis ofvariance using only intervention and control participants who attended the 4 month follow up. All models were adjusted for the baseline age and sex. McNemar test was utilized to compare the proportions of behaviour changes of and assessed for the trends of changes. BMI z-score and body fat percentage of intervention participants at baseline, 4 months and 8 months were compared pair-wisely using tests of within subject contrasts in repeated measures ANOVA to assess for program sustainability with adjustment for baseline age and sex.

## Results

Among the 165 subjects, 80 and 85 were randomised to the intervention and control arms. Some students had other prior committed extra-curricular activities clashing with time schedule of this programme so 106 subjects finally enrolled in the study with 57 subjects in the intervention groups. Some students had other prior committed extra-curricular activities clashing with time schedule of this programme so only 106 subjects finally enrolled in the study. The numbers of students finally in intervention and control groups were not markedly different. They did not leave the study due to disappointment. It is not uncommon in Hong Kong that students enroll in many activities at different time points.

Fifty three subjects completed the 4-months intervention program and 43 (75%) and 51 (89%) attended the 4-month and the 8-month follow up respectively. Forty-nine participants in the control arm attended the baseline assessment, 41 (84%) were assessed at 4 month and 45 (92%) at 8 month. There were no significant differences between participants who attended follow up (n = 79) compared with those not attending (n = 27) with regard to sex, age, BMI z-score or body fat percentage. No differences were found between study groups in terms of socio-demographic and anthropometric characteristics (body fat percentage and BMI z scores) at baseline except the control group were older and had a higher BMI compared to the intervention group (Table 2). Age difference has been adjusted in both intention-to-treat and completer analysis. Measure of relative BMI has been adjusted for the child's age and sex using BMI z-score, a preferred indicator for evaluating treatment success in longitudinal studies. No adverse events were reported during the intervention.

**Table 2:** **Baseline characteristics of study participants according to group** ^a^

Variable	All randomised participants (ITT)			Completers		
	Intervention group	Control group	P	Intervention group	Control group	P
	(n =57)	(n =49)		(n =43)	(n =40)	
Male, number (%)	38 (66.7)	37 (75.5)	0.318	30 (70)	29 (70)	0.982
Obese, number (%)	21 (36.8)	23 (46.9)	0.293	15 (35)	17 (43)	0.476
Age, year	10.1(0.9)	10.7(1.0)	0.001	10.0 (0.9)	10.6 (1.0)	0.006
BMI, kg/m^2^	23.6 (2.3)	25.3 (3.2)	0.002	23.6 (2.6)	24.9 (2.9)	0.027
BMI z-score	1.76 (0.32)	1.88 (0.34)	0.076	1.76 (0.35)	1.84 (0.33)	0.247
Body fat, %	30.4 (5.4)	32.1 (6.9)	0.158	30.5 (5.4)	32.2 (6.7)	0.214
Waist to height ratio	0.57 (0.04)	0.59 (0.05)	0.047	0.57 (0.04)	0.59 (0.04)	0.118

^a^Data are presented as mean (standard deviation) unless otherwise indicated.

ITT – intention-to-treat analysis, BMI- body mass index.

### Primary outcomes

Table 3 shows the changes in BMI z-score and body fat percentage between groups and within groups at the completion of active intervention program. Intention-to-treat analyses revealed that the intervention group significantly reduced their BMI z-score (-0.21, 95% CI -0.34 to -0.07, P = 0.003) and body fat (-2.67%, 95% CI -5.12 to -0.22, P = 0.033) compared to control group. For those completers, intervention group also had significant reduction in BMI z score (-0.16, 95% CI -0.3 to -0.02) and body fat (-3.09%, 95% CI -5.91 to -0.26). Table 4 shows the change in primary outcomes in intervention group at 8 months from baseline. After 4 months maintenance period, significant reduction in BMI z-score (-0.06, 95% CI -0.11, -0.007, P = 0.028) and to a lesser extent body fat (-1.71%, 95% CI, -3.44 to 0.02, P = 0.052) was observed in the intervention group. Completer analyses also showed similar results.

**Table 3:** **Changes in primary outcomesat 4 months from baseline** ^a^

	All randomised participants			Completers		
	Intervention group (n = 57)	Control group (n = 49)	Group difference ^1^ (intervention minus control)	Intervention group (n = 43)	Control group (n = 40)	Group difference ^2^ (intervention minus control)
BMI z-score	0.02 (-0.17 to 0.21)	0.48** (0.25 to 0.71)	-0.21* (-0.34 to -0.07)	-0.04** (-0.01 to -0.08)	0.02 (-0.03 to 0.06)	-0.16* (-0.30 to -0.02)
Body fat (%)	0.40 (-0.56 to 1.37)	1.92** (0.72 to 3.13)	-2.67* (-5.12 to -0.22)	0.54 (-0.76 to 1.83)	2.36** (0.92 to 3.80)	-3.09* (-5.91 to -0.26)

^a^Data are presented as mean (95% confidence interval).

^1^Linear mixed model, adjusted for baseline age and sex.

^2^Repeated measure of ANOVA, adjusted for baseline age and sex.

*P < 0.05.

**P < 0.005.

**Table 4:** **Changes in anthropometric outcome variables of intervention group at 8 months from baseline** ^**1**^ **(n = 41)**

	Change 4–8 months		Change 0–8 months	
	Mean (95% CI)	P	Mean (95% CI)	P
BMI z-score	-0.06 (-0.11 to -0.01)	0.028	-0.11 (-0.17 to -0.005)	0.001
Body fat (%)	-1.71 (-3.44 to 0.02)	0.052	-1.26 (-2.6 to 0.85)	0.066

^1^Repeated measure of ANOVA, adjusted for baseline age and sex.

### Dietary behaviours

At 4 months follow up, higher proportion of participants consumed healthy food, lower proportion of participants consumed unhealthy food, and eating habits were better controlled (Table 5). The improvement would be maintained or even better at 8 months follow up. Reverse trend was reported for the control group. Although no statistical significant differences were detected, the results showed opposite trend with intervention group being more positive and control group being more negative. The results of parent survey among 48 students from intervention groups also showed similar trends.

**Table 5:** **Self-reported dietary and exercise behaviourat Baseline and 4 months in intervention and control group students**

	Intervention group			Control group			P value ^1^
	(n =43)			(n =29)			
	Percentage			Percentage			
	baseline	4 months	Change	baseline	4 months	Change	
			(95% CI)			(95% CI)	
**Fruit and vegetables**							
Fruit ≥1 serve/day	40	42	+*2* (-25 to 3)	55	55	0.0 (-38 to 38)	1.000
Vegetables ≥2 serves/day	35	44	+*9* (-17 to 36)	31	21	*-10* (-36 to 16)	0.481
**High fat food**							
Processed meat ≥4 times	29	12	*-17* (-35 to 2)	24	38	+*14* (-42 to 14)	0.118
Deep fried food ≥4 times	19	7	*-12* (-26 to 3)	7	10	+*3* (-12 to 19)	0.227
Crisp ≥4 times	12	2	*-9* (-20 to 2)	3	10	+7 (-6 to 20)	0.219
**High sugar food and beverage**							
Sugary beverage ≥4 times	33	21	-12 (-33 to 10)	35	24	-10 (-38 to 17)	0.302
Dessert ≥4 times	9	0	*-9* (-18 to -1)	3	7	+*4* (-8 to 15)	0.453
Sweet or chocolate ≥4 times	14	7	*-7* (-20 to 7)	3	7	+*4* (-8 to 15)	0.508
**Self-control**							
Avoid overeating	14	28	+*14* (-5 to 33)	24	24	*0.0* (-25 to 25)	0.146
**Exercise**							
30-mins light intensity exercise ≥3 days	24	29	+5 (-17 to 27)	45	41	*-3* (-37 to 3)	0.774
60-min moderate intensity exercise ≥3 days	31	33	+2 (-22 to 27)	28	35	+7 (-22 to 35)	1.000
20-min aerobic exercise ≥3 days^#^	41	45	+*5* (-23 to 33)	45	31	*-14* (-45 to 18)	0.804
Strengthening exercise ≥3 days^#^	12	26	+14 (-4 to 32)	7	21	+14 (-5 to 32)	0.109
I like exercise	61	91	+*30* (-5 to 66)	62	69	+*7* (-35 to 46)	0.002*
I have excuses for not doing exercise	14	9	*-5* (-19 to 10)	7	17	+10 (-7 to 28)	0.727
I am fear of sport injury	19	12	*-7* (-23 to 9)	7	10	+*3* (-12 to 19)	0.375

^1^P-values are for the change between 3 and 6 months by the McNemar’s Test.

*Italic* highlights the effect size in opposite direction or statistical significance at level of P 0.05*.

The evaluation results also showed that parents adopted healthier cooking methods with fewer parents using deep-frying in cooking from 12.5% at T_0_ to 7.1% at T_1_ and 1.8% at T_2_.

### Exercise habits

At 4 months follow up, proportion of students fond of exercise increased from 61% to 91% (P = 0.002) in the intervention group (Table 5). Fear of sport injury dropped in the intervention group and reverse pattern was observed in the control group. For self reported exercise, proportion of students participating in different types of exercise increased in the intervention group but the proportion decreased in the control group except 60 minute moderate intensity exercise with (Table 5). Similarly continuous improvement trend was observed for performing strengthening exercise, aerobic exercise and moderate intensity exercise at 8 months follow up. The results were consistent with the findings from parents’ survey. More parents (+26.5% T_1_ to T_0_ P = 0.02) reported that their children were eager to do more exercise. About 50% of parent reported that their children increased participation in sports activities from T_0_ to T_2_. A significant higher proportion of parents encouraged their children to engage in sports (+34% from T_0_ to T_1_, P < 0.001), and discussed the benefit of exercises and negative effects of physical inactivity with their children (+27.7% T_0_ to T_1_, p = 0.004). A significant higher proportion of parents praised their children for doing exercise (+27.7% from T_0_ to T_1,_ p = 0.004) and bought sports equipment for their children (=25.6% from T_0_ to T_1,_ p = 0.002). More parents also reported doing 20 minutes exercise with their children at least once per week (+18.2% from T_0_ to T_1)._

### Impact of project on empowering schools to foster a supportive healthy living environment

Participating schools run various school-based health-promoting programmes ranging from 5 to 34 activities for students and their parents under project team support and guidance during the project period. Out of a total of 10 scores, participating schools rated an average of 8.07 for sufficiency of support from project team. Mean score was 8.67 for agreeing the programme enhanced the teachers’ competency in running school-based weight management programme and understanding of the fitness status of students, and broaden the school’s horizon in health policy and education.

### Programme feedback from students and parents

A total of 67 feedback forms were returned from students of both intervention and control groups. Majority of students expressed that the programme had enhanced their knowledge on healthy eating and exercise for weight control, and also raised their interest in healthy eating and sport activities. Nearly 70% of students expressed their wishes to have similar programme in future. For overall rating of the programme, the mean score was 7.27 out of total of 10 with SD ± 2.44.

A total of 77 feedback forms were returned from parents. Over 90% reported that the programme had enhanced their knowledge in helping their children eating healthily and exercising for weight control as well as arousing their awareness on importance of weight control, and increased their understanding on the fitness status of their children. Over 70% of the parents felt that the programme had posed positive influence to their children’s self-esteem and also parent–child relationship. The mean score rated by parents was 7.89 out of a total of 10 with SD ±1.48.

## Discussion

This pilot trial study addressed the need of focusing on a weight management programme for overweight and obese children delivered in school setting. To the best of our knowledge, it is one of the first weight management trial evaluated in a school setting versus a clinical or community setting. We demonstrated that a 4-month school-based child weight management programmes delivered in HPS is efficacious for obesity management after intervention and at 8-month follow up. Significant changes in BMI z-score and percent body fat were observed. The reduction in BMI z-score are of similar magnitude as reported in 2009 Cochrane review of lifestyle trials in children under 12 years old [37]. Although statistical significant improvements were observed only on few health behaviours with established linked to weight control, reverse trend was observed for control group. The findings still alert the possible impact on behavioural change.

Integration of knowledge concerning healthy dietary intake, physical activity habits, and smoking and drug prevention through specified modified school curriculum needs to act in parallel with other strategies to tackle the environmental and economic limitations [28]. An Active Programme Promoting Lifestyle Education in School in England [40, 41] and some previous studies mainly showed change in nutritional knowledge rather than significant changes in behaviour or short to intermediate term health outcomes [42, 43]. Cost-effective intervention should target children at higher risk and devote more resources to intensive interventions integrating nutrition and physical activities with strong family components similar to this study and some other recent studies. [23, 43].

The WHO concept of HPS is seen as most promising approach as it incorporates actions addressing the school physical and social environment not just health education. The interventions adopted in this study were embedded in a supportive environment for HPS. If the targets were weight loss and weight maintenance, the insignificant findings of wait-list schools despite the supportive environment suggest that the HPS model ought to be supplemented by intensive interventions and parental engagement. The researchers of this study have successful implemented HPS in Hong Kong demonstrating health improvement amongst students [23, 29]. This programme building on the successful model of HPS has demonstrated the potential of overcoming the environmental and economic barriers, and moving beyond curriculum as curriculum approach alone had limited effectiveness in improvement of physical activities. The school environment would shape youth health via common pathways [44]. This might explain why not many other school based weight control programmes showed improvement of anthropometric measures within a short period of time. Building on existing HPS framework would add synergy to school based health promotion initiatives.

There are limitations of this pilot study. We are unable to determine if the environment component was essential to success because of design limitations in this pilot study. Further research should compare students receiving group education/counselling and parent education alone with students receiving the intervention within boarder HPS activities. The sample size of this study might not have adequate power to detect the difference of behaviours between intervention and control groups with statistical significant. Behaviour outcomes should also be quantified more precisely. It was not done in this pilot study to avoid over burdening the participants. Statistical significant difference of mean age 10.1 vs 10.7 was found between intervention and control groups but the impact on health behaviours would be minimal. Although the programme has received very positive feedbacks from students, parents and schools, the duration of intervention was too short for monitoring longer term effects. The heavy commitment of students in other activities also limited the participation of eligible students in this programme. Education sector should take note of the findings and evaluation from stakeholders of this pilot programme and accord higher priority for school based weight management programme.

Our programme puts a strong emphasis on parental participation with more parents engaging in exercise, improved cooking styles and discussing the importance of physical activities with their children, and continued to do so after intervention period. A systematic review of environmental correlates of obesity-related youth dietary behaviours shows consistent associations between parental intake and children’s fat and their fruit/vegetable intakes [45]. Long term school-parent partnership and education intervention has shown positive statistical significant results in overall physical activity incidence, and adiposity and fitness measures such as BMI, sit and reach flexibility, and 20 m shuttle run [46]. It would also help families at lower socio-economic status to ameliorate the moderation effect of socio-economic status on relationship between psychosocial predictors and healthy eating of students [47].

## Conclusion

The scale of the obesity epidemic makes it difficult to manage overweight and obese children on one to one basis through clinical services alone. This study has demonstrated the efficacy of school-based weight management programme targeting overweight and obese children with intensive education and counselling, and parental engagement operating within a broader HPS approach. The programme needs to incorporate socio-environmental factors, personal factors and behavioural factors putting the issue in the context of everyday life [48], and strategies based exclusively on diet may not stop weight gain [23].

## Electronic supplementary material

Additional file 1: **Intervention at school level and strategies to involve the parents.** (PDF 212 KB)

Additional file 2: **Students’ dietary behaviour in the week prior to the survey at T0, T1 and T2.** (PDF 154 KB)
