# Supplementary material for: Childhood obesity management shifting from health care system to school system: intervention study of school-based weight management programme
Source: BMC Public Health. 2014 Nov 3;14:1128. doi: 10.1186/1471-2458-14-1128 (PMC4289207; doi:10.1186/1471-2458-14-1128)
Supplement: Supplementary file 1 — Additional file 1: Intervention at school level and strategies to involve the parents. (PDF 212 KB) [file 12889_2013_7412_MOESM1_ESM.pdf]

Additional file 1 – Intervention at school level and strategies to involve the parents

1. Empowerment and Supports for Teachers

Each of the participating school was guided to form a teacher working team to work closely with the project team and they were required to submit an annual plan of school-based health promoting activities during the project period. Teachers were valued as key person in program development and implementation. Advice and guidance were given to individual schools by the project dietitian and physiotherapist throughout the project period. In addition, 3 workshops and discussion sessions were organised for the teachers with the objectives to:

- Empower teachers with confidence and skills in preventing and managing childhood obesity; in fostering a supportive school environment facilitating students to develop healthy eating habits and active lifestyle;
- Review the progress and effectiveness of the project; and
- Share among the participating schools on problems encountered during implementation and explore for possible solutions.

2. School-based Health Promotion Programme

The participating schools were guided and supported by the project team to carry out a series of school-based activities promoting healthy eating and active lifestyles throughout the project period to enhance awareness of health lifestyle to participating students as well as the rest of students in school. Examples of healthy eating promotion activities organised by schools included:

- fruity month;
- vegetarian day;
- healthy tuck shop scheme;
- healthy snack award scheme;
- healthy cooking competition;
- healthy breakfast day.

Examples of exercise promotion activities organised by schools included;

- morning and recess exercise session;
- morning walking scheme;
- “Sports Act” Award scheme;
- rope jumping campaign;
- mini Olympic games at school.

Other health promotion activities included health ambassador scheme, healthy poster design competition, healthy-theme classroom decoration, health carnival, etc. Many of the initiatives invited the participation of family members and teachers.
