# Supplementary material for: Childhood obesity management shifting from health care system to school system: intervention study of school-based weight management programme
Source: BMC Public Health. 2014 Nov 3;14:1128. doi: 10.1186/1471-2458-14-1128 (PMC4289207; doi:10.1186/1471-2458-14-1128)
Supplement: Supplementary file 2 — Additional file 2: Students’ dietary behaviour in the week prior to the survey at T0, T1 and T2. (PDF 154 KB) [file 12889_2013_7412_MOESM2_ESM.pdf]

Additional file 2

Students' dietary behaviour in the week prior to the survey at T_0, T_1 and T_2

Fruit and Vegetables

Fruit ≥ 1 serve/day

Vegetables ≥ 2 serves/day

High Fat Food

Processed or preserved meat ≥ 4 times

Deep fried food ≥ 4 times

Crispy food ≥ 4 times

High Sugar Food and Beverage

Sugary beverage ≥ 4 times

Dessert ≥ 4 times

Sweet or chocolate ≥ 4 times

Self Control

Avoid overeating

Removed fat or skin from meat or poultry

Students' exercise habit in the week prior to the survey at T_0, T_1 and T_2

30-min light intensity exercise ≥ 3 days

60-min moderate intensity exercise ≥ 3 days

20-min aerobic exercise ≥ 3 days

Strengthening exercise ≥ 3 days

Students' attitude towards exercise at T_0, T_1 and T_2

I like exercise

I have excuses for not doing exercise

I am fear of sport injury

My parents have discussed about negative effects of inactivity with me

Parent reported exercise habit of their children at T_0, T_1 and T_2

My child is eager to participate in sport activities

My child does housework with me every day

Self-esteem of students at T_0, T_1 and T_2

I am satisfied with myself on the whole

I have a number of good qualities

Proportion of parents reported deep-frying as a commonly used home cooking method at T_0, T_1 and T_2

Deep-frying was a commonly used cooking method at home

Parents' action to encourage children in sport participation at T_0, T_1 and T_2

Encouraged children to engage in sport

Told children about the benefits of exercise and the negative effects of inactivity
Praised children for doing exercise

Bought sports equipment to children

Exercise with children at least 20 minutes ≥ 1 times/wk
